# Oxidative Stress and Alterations in the Antioxidative Defense System in Neuronal Cells Derived from NPC1 Patient-Specific Induced Pluripotent Stem Cells

**DOI:** 10.3390/ijms21207667

**Published:** 2020-10-16

**Authors:** Alexandra V. Jürs, Christin Völkner, Maik Liedtke, Katharina Huth, Jan Lukas, Andreas Hermann, Moritz J. Frech

**Affiliations:** 1 Translational Neurodegeneration Section Albrecht Kossel, Department of Neurology, University Medical Center Rostock, University of Rostock, 18147 Rostock, Germany; alexandra.juers@uni-rostock.de (A.V.J.); Christin.Voelkner@med.uni-rostock.de (C.V.); Maik.Liedtke@med.uni-rostock.de (M.L.); Katharina.Iwanov@med.uni-rostock.de (K.H.); jan.lukas@med.uni-rostock.de (J.L.); Andreas.Hermann@med.uni-rostock.de (A.H.); 2 Center for Transdisciplinary Neurosciences Rostock (CTNR), Rostock University Medical Center, University of Rostock, 18147 Rostock, Germany; 3 German Center for Neurodegenerative Diseases (DZNE) Rostock/Greifswald, 18147 Rostock, Germany

**Keywords:** NPC1, ROS, catalase, SOD1, SOD2, iPSC

## Abstract

Oxidative stress (OS) represents a state of an imbalanced amount of reactive oxygen species (ROS) and/or a hampered efficacy of the antioxidative defense system. Cells of the central nervous system are particularly sensitive to OS, as they have a massive need of oxygen to maintain proper function. Consequently, OS represents a common pathophysiological hallmark of neurodegenerative diseases and is discussed to contribute to the neurodegeneration observed amongst others in Alzheimer’s disease and Parkinson’s disease. In this context, accumulating evidence suggests that OS is involved in the pathophysiology of Niemann-Pick type C1 disease (NPC1). NPC1, a rare hereditary neurodegenerative disease, belongs to the family of lysosomal storage disorders. A major hallmark of the disease is the accumulation of cholesterol and other glycosphingolipids in lysosomes. Several studies describe OS both in murine in vivo and in vitro NPC1 models. However, studies based on human cells are limited to NPC1 patient-derived fibroblasts. Thus, we analyzed OS in a human neuronal model based on NPC1 patient-specific induced pluripotent stem cells (iPSCs). Higher ROS levels, as determined by DCF (dichlorodihydrofluorescein) fluorescence, indicated oxidative stress in all NPC1-deficient cell lines. This finding was further supported by reduced superoxide dismutase (SOD) activity. The analysis of mRNA and protein levels of SOD1 and SOD2 did not reveal any difference between control cells and NPC1-deficient cells. Interestingly, we observed a striking decrease in catalase mRNA and protein levels in all NPC1-deficient cell lines. As catalase is a key enzyme of the cellular antioxidative defense system, we concluded that the lack of catalase contributes to the elevated ROS levels observed in NPC1-deficient cells. Thus, a restitution of a physiological catalase level may pose an intervention strategy to rescue NPC1-deficient cells from the repercussions of oxidative stress contributing to the neurodegeneration observed in NPC1.

## 1. Introduction

The disease Niemann-Pick type C1 (NPC1) is a rare neurovisceral lysosomal storage disorder that is caused by autosomal recessive mutations in the *NPC1* gene. Due to the reduced function of the cholesterol transporter NPC1, mainly unesterified cholesterol, as well as other types of lipids like gangliosides and sphingosines, accumulate primarily in lysosomal/late-endosomal compartments [1]. Varying in age of onset and severity of the clinical presentation, NPC1 is characterized by neurodegeneration and a hepatosplenic phenotype [1,2]. While the transport mechanism of cholesterol to the mitochondria is still not fully understood, an increased amount of cholesterol in the mitochondria of NPC1-deficient cells has been demonstrated [3,4]. This leads to mitochondrial dysfunction and alterations in the electron transport chain [3,5,6,7,8], ultimately promoting the production of reactive oxygen species (ROS). Such a disbalance of ROS production and elimination, leading to an increase in ROS, is defined as oxidative stress (OS) [9]. OS is a common pathophysiological feature in neurodegenerative diseases and has also been described for NPC1. ROS directly attack biomolecules and induce permanent cellular damage, often leading to apoptosis [10,11,12]. In this context, increased ROS levels were found in a number of NPC1 in vivo and in vitro models, ranging from rats treated with NPC1 inhibitor U18666A, BALB/c mice carrying either different NPC1 mutations or a knockout, a yeast homolog of a NPC1 model and CHO cells to human neuroblastoma cells and NPC1 patient-specific fibroblasts [13,14,15,16,17].

In a NPC1 mouse model, both increased ROS levels and elevated nitro-tyrosine fractions were detected in the hippocampus, the thalamus and the cerebellum [14,18,19]. Additionally, oxidized biomolecules were found in these NPC1-deficient mice [7,20]. Comparable results were obtained from the hippocampus of rats treated with U18666A, wherein DCF (dichlorodihydrofluorescein) fluorescence was used to detect increased ROS levels and supported by nitro-tyrosine stainings [14]. Alterations in the antioxidative defense system, including diminished mitochondrial glutathione (mGSH) and variations in superoxide dismutase (SOD) levels, were found [6,7]. Moreover, elevated proapoptotic proteins like c-Abl/p73 were detected in NPC1-deficient mice, demonstrating that OS contributes to the induction of apoptosis [21]. 

Similar hallmarks of OS can be detected in different in vitro models. In NPC-deficient neuroblastoma cells, higher ROS levels were detected by flow cytometry measurements [13,22]. Dominko and colleagues [22] pointed out differences in the defense system of the NPC1-deficient CHO cells represented by lower GSH content, decreased catalase activity and elevated SOD activity. Similarly, SOD alterations, as well as differences in the glutathione transferase (GST), were found in the cerebrospinal fluid of NPC1 patients [23]. In addition, CoQ10 deficiency and reduced antioxidant capacity causing elevated lipid peroxidation were observed in the plasma of NPC1 patients [24,25]. Studies using NPC1 patient-derived fibroblasts carrying various mutations of the NPC1 gene revealed higher ROS levels and increased markers of oxidized biomolecules [13,16], as well as alterations in the antioxidative defense system like the impaired expression of genes that are related to ROS production and decreased catalase activity [5,13,26,27].

Despite the variety of models used so far, studies employing NPC1 patient-derived cells other than fibroblasts are limited. This holds especially true for human NPC1-deficient neurons. Thus, we were interested in different hallmarks of OS displayed by human neuronal cells. In this study, we investigated OS parameters in neuronal cells derived from NPC1 patient-specific induced pluripotent stem cells [28]. These cells were analyzed regarding the alterations of ROS levels and SOD activity and the amount of nitrated proteins, as well as the expression of SODs and catalase.

## 2. Results

### 2.1. NPC1-Deficient Cells Display Hallmarks of Oxidative Stress

Increased levels of ROS and associated changes in cellular pathways and functions contribute crucially to the pathology of neurodegenerative diseases such as NPC1 [8,29]. Elevated ROS levels were detected in several NPC1-deficient cell types, but data from NPC1-deficient human neuronal cells are missing. Thus, we determined the intracellular ROS level of neuronal derivatives of human patient-specific induced pluripotent stem cells (iPSCs) in control and NPC1-deficient cells.

**Figure 1:**
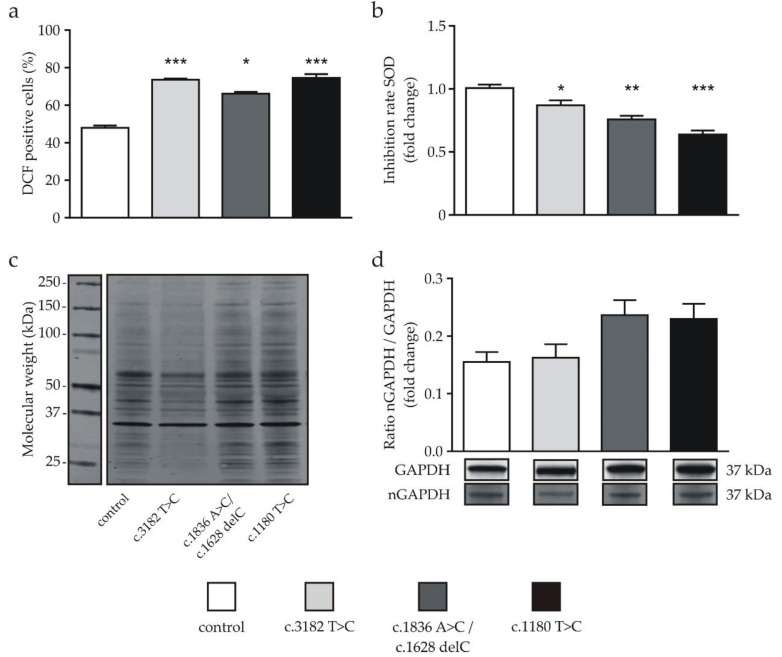
Determination of oxidative stress markers in neuronal differentiated cells. (**a**) Significantly increased reactive oxygen species (ROS) levels of Niemann-Pick type C1 (NPC1)-deficient cell lines in comparison to the control cell line detected by the flow cytometry analysis of DCF fluorescence (*n* = 16–18). (**b**) Significantly decreased superoxide dismutase (SOD) activity was observed in the NPC1-deficient cells compared to the control (*n* = 10–22). (**c**) Determination of nitrated proteins as an oxidative stress marker. NPC1-deficient cell lines displayed distinct patterns of bands with a molecular weight below 150 kDa and above ~50 kDa. An example of complete gel is shown Supplementary Figure S2. (**d**) The ratio of nitrated proteins located at the 37-kDa (nitrated glycerinealdehyd-3-phosphat-dehydrogenase (nGAPDH)) band and the amount of GAPDH (GAPDH) was increased in two NPC1-deficient cell lines; the amount of nitrated GAPDH was not significantly different (*n* = 11–21). Cropped bands are shown as an example and display corresponding examples of the same gel (*n* = 10–26). * = *p* < 0.05, ** = *p* < 0.01 and *** = *p* < 0.001. Statistical tests used: (**a**) one-way ANOVA with Kruskal-Wallis multiple comparison test, (**b**,**d**) ordinary one-way ANOVA with Dunnett’s multiple comparisons test. See also the Materials and Methods section.

ROS were measured using the fluorescent dye DCF, which is formed from H_2_DCF upon contact with ROS. In addition, we determined propidium iodide (PI)-positive cells reflecting necrotic or apoptotic cells in the examined population. Only PI-negative cells and DCF-positive cells displayed in the lower right quadrant (Supplementary Figure S1a,b) were included in the evaluation. In accordance with recent studies [13,14], we found significantly higher ROS levels in each of the NPC1-deficient cell lines compared to the control (Figure 1a). As a further indicator of OS, SOD activity was significantly decreased in all NPC1-deficient cell lines in comparison to the control cell line (Figure 1b). Western blot analysis revealed a distinct pattern of proteins with nitrated tyrosine residues (Figure 1c). Although the identity of these particular proteins remains to be determined, the band pattern of nitrated proteins differs between the cell lines (Figure 1c), indicating that NPC1-deficient cells suffer from oxidative stress. In the control and the c.3182 T > C cells, the majority of the bands were detected between 37–75 kDa, while, in the cell lines c.1836A > C/c.1628 delC and c.1180 T > C, the bands were more pronounced and spread out from 20 to 200 kDa. To quantify the amount of nitrated proteins, we analyzed glycerinealdehyd-3-phosphat-dehydrogenase (GAPDH), which is known to undergo nitration of tyrosine residues [30]. We found a higher ratio of nitrated GAPDH (nGAPDH) to total GAPDH in the cell lines carrying the c.1836 A > C/c.1628 delC and c.1180 T > C mutations, although the differences were not statistically significant (Figure 1d).

As we have shown that NPC1-deficient cells suffer from OS, we next investigated changes of the antioxidant enzymes. Thus, we determined the gene expression of two isoforms of superoxide dismutase (SOD), namely SOD1 and SOD2, as well as catalase, representing key players of the antioxidant system.

### 2.2. Antioxidant Response mRNAs are Differentially Regulated in NPC1 Mutants

**Figure 2:**
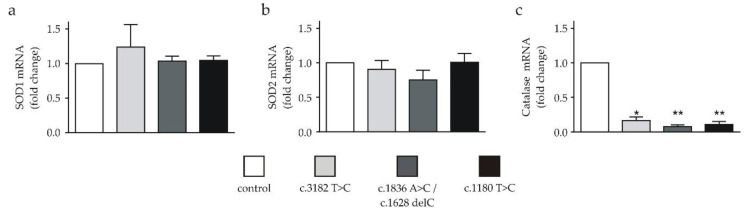
mRNA expression of SOD1, SOD2 and catalase in neuronal differentiated cells. (**a**) SOD1 mRNA and (**b**) SOD2 mRNA expressions in NPC1-deficient cell lines were not different in comparison to the control (*n* = 5 or 6). (**c**) All NPC1-deficient cell lines showed a significantly lower expression of catalase mRNA (*n* = 6). * = *p* < 0.05 and ** = *p* < 0.01. Statistical tests used: (**a**–**c**) one-way ANOVA with Kruskal-Wallis multiple comparisons test. See also the Materials and Methods section.

Recent studies employing NPC1-deficient fibroblasts [5] or NPC1-deficient mice [6] described alterations in the expression of genes involved in OS. To evaluate the selected gene expression changes in our neuronal NPC1 cell model, we investigated SOD1, SOD2 and catalase, representing important antioxidative defense components. Unexpectedly, none of the NPC1-deficient cell lines displayed an altered SOD1 or SOD2 mRNA expression (Figure 2a,b). Interestingly, a significant reduction in catalase mRNA was detected in all NPC1-deficient cell lines in comparison to the control cell line (Figure 2c).

### 2.3. Protein Expression of Antioxidant Enzymes in NPC1-Deficient Cell Lines

**Figure 3:**
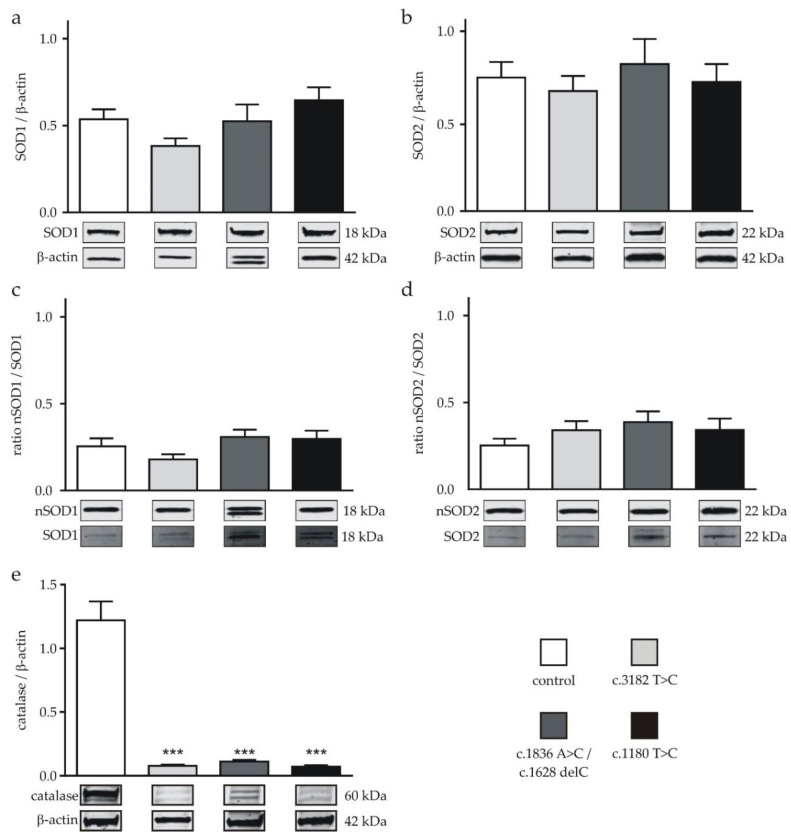
Protein expression of SOD and nitrated (n)SOD and catalase in neuronal differentiated cells. (**a**) Expression of SOD1 and (**b**) SOD2 of all NPC1-deficient cell lines was not different to the corresponding amounts in the control cell line (*n* = 10–13). (**c**) The ratio of the nitrated proteins located at the 22-kDa (nSOD1) band to the total SOD1 and (**d**) the ratio of nitrated proteins located at the 22-kDa (nSOD2) band to the total SOD2 were similar in all cell lines (*n* = 4–13). (**e**) The expression of catalase was significantly lowered in all NPC1-deficient cell lines (*n* = 10–13). Double bands, observed for catalase, most likely represent isoforms of the protein. Western blot bands display corresponding examples of the same gel. An example of a complete gel is shown in Supplementary Figure S3. *** = *p* < 0.001. Statistical tests used: (a–e) ordinary one-way ANOVA with Dunnett´s multiple comparisons test. See also the Materials and Methods section.

Next, we analyzed the expressions of SOD1, SOD2, the ratio of nitrated fractions (nSOD1 and nSOD2) and catalase by Western blot to reveal correlations between the mRNA levels and protein levels of the corresponding enzymes. In none of the cells carrying a NPC1 mutation, we found a significant alteration of the SOD1 or SOD2 protein levels (Figure 3a,b). The same observation was made for the ratio of nSOD1 and nSOD2 to the respective total protein amount. None of the NPC1-deficient cell lines displayed an altered ratio in comparison to the control (Figure 3c,d).

Since we detected a reduced catalase mRNA level, we suspected a reduction of the protein level. Indeed, we found a significantly decreased catalase level in all NPC1-deficient cell lines, wherein the protein amount was reduced by ~90% of the amount of the control cell line in the NPC1-deficient cells (Figure 3d).

Contrary to our expectations, based on the observed pattern of nitrated proteins and nitrated GAPDH (Figure 1c,d), neither the SOD1 nor the SOD2 protein expression was altered in the NPC1-deficient cells. These findings suggest an impact on the SOD activity, without a change in the overall protein level.

In summary, we demonstrated for the first time that neuronal cells derived from NPC1 patient-specific induced pluripotent stem cells suffer from oxidative stress. Higher ROS levels, determined by DCF fluorescence, as well as a reduced SOD activity and slightly changed amounts of nitrated proteins, indicate OS in all NPC1-deficient cell lines. Transcription and translation of the most common proteins of the superoxide and hydrogen peroxide metabolism, namely SOD1 and SOD2, were unaltered, but we observed a striking reduction of the mRNA and protein level of catalase.

## 3. Discussion

Oxidative stress is a common feature in neurodegenerative disorders, and growing evidence suggests that OS is involved in the pathophysiology of Niemann-Pick type C1 disease (NPC1). OS has been shown for a variety of NPC1-deficient in vitro model systems, like patient-specific fibroblasts or murine models [13,23]. To our knowledge, no studies analyzing OS in a human neuronal model system are available so far. Thus, we investigated the level of OS and the alterations of the antioxidative defense system in a human cell model based on neuronal differentiated cells (NDCs) derived from NPC1 patient-specific induced pluripotent stem cells [23,28,31,32].

The elevated ROS level observed here is indicative of OS and is in accordance with results of studies with the widely used BALB/c NPC1-deficient mouse strain [14], NPC1-deficient fibroblasts [13] and NPC1-deficient CHO cells [15]. Another sign of OS is an increased level of nitrated proteins. The reaction of nitric oxide and superoxide leads to the production of peroxynitrite. This radical directly attacks biomolecules like proteins, and a carbon-nitrogen bond is formed at tyrosine residues [33]. In accordance with findings in the cerebellum [7,18] and in the thalamus [19,34] of murine NPC1 models, we observed a slightly elevated level of nitrated proteins in the NPC1-deficient neuronal differentiated cells (NDCs). In contrast to these former studies, in which immunofluorescence stainings were used, we investigated the nitro-tyrosine level using Western blot analysis. Klein and colleagues described two bands containing nitrated proteins in the cerebellum of a murine NPC1 model [14]: one ranging from 120 to 35 kDa and another one ranging from 30 to 18 kDa. In contrast, we detected several distinct bands in the Western blot of the cell lysate obtained from NDCs. Cells carrying the abnormal alleles c.1836 A > C/c.1628 delC in compound heterozygosity and a homozygous allele variant, c.1180 T > C, displayed a different pattern of bands, containing more bands with a higher content of nitrated proteins. The differences in the observed band patterns in the study of Klein and colleagues and our study, as well as the identity of nitrated proteins, remain elusive. However, we speculate that this observation is based on an elevated ROS level in NPC1-deficient cells. As an example of a protein that is known to undergo nitration, we analyzed the ratio of nitrated GAPDH (nGAPDH) to GAPDH [30,35]. The increased ratio of nGAPDH/GAPDH in at least two NPC1-defcient cell lines suggests that the cells suffer from OS.

Cells can respond to OS with a variety of antioxidant enzymes. SOD is discussed to be a key player in the regulation of the cellular ROS levels, since it mediates the dismutation of superoxide radicals into hydrogen peroxide and molecular oxygen [36], which, in turn, can be eliminated by glutathione peroxidases, catalase and peroxiredoxins [11]. SOD activity has been studied in the context of NPC1 before, with inconsistent results. While no changes in the SOD activity were observed in NPC1 patient erythrocytes [25], a decreased SOD2 activity was described for ncr1Δ yeast cells, although the total SOD activity was not altered in this yeast model [37]. In contrast, our iPSC-based neuronal cell model showed a decreased SOD activity in all cell lines carrying a NPC1 mutation. As we have demonstrated that proteins of NPC1-deficient cells undergo nitration, and since SOD2 is a main target for the nitration of tyrosyl residues in the active center of the protein [35,38,39], we determined the ratio of nitrated SOD1 (nSOD1) and total amount of SOD1 (nSOD1/SOD1), as well as the nSOD2/SOD2 ratio, in NPC1-deficient cells. In none of the three NPC1-deficient cell lines, we found an elevated ratio of nSOD1/SOD1 or nSOD2/SOD2 (Figure 3). Thus, one could conclude that the inhibition of SOD activity is not based on nitration of the protein. On the other hand, we observed a slightly, but statistically not significant, increase of nSOD2/SOD2, suggesting that nitration may impair SOD activity to some extent.

To further investigate the SOD status in the NPC1-deficient cells used here, reverse transcription quantitative real-time polymerase chain reaction (RT-qPCR) and Western blot analysis were performed to reveal alterations in the expressions of SOD1, SOD2 and catalase. SOD2, also known as Mn-SOD, is located in the mitochondrial matrix and is of high relevance for the regulation of oxidative stress and cell survival [36]. Several studies have shown alterations of SOD2 protein and mRNA expression levels in NPC1 models before, with varying results. In a murine model, an increased SOD2 expression was detected in lysates of the cerebellum [6], as well as in the cerebellar Purkinje cells [16]. In contrast, a study with NPC1-deficient mice described a decreased SOD2 gene expression in neural stem cells [40]. In NPC1 patient-derived fibroblasts, downregulation of the gene expression and a diminished protein level of the SOD2 were also found [26,27]. However, Dominko and colleagues [22] found changes neither of the SOD1 nor the SOD2 protein level in NPC1-deficient CHO cells, although they detected changes in SOD activity [22]. Unexpectedly, we did not observe any alterations in the regulation or expression of SOD1 and SOD2. This result is in contrast to studies describing a downregulation of SOD2 by epigenetic silencing [41] or a higher SOD2 gene expression triggered by increased ROS levels [36]. A transcriptional induction might not be visible on the protein level, given that tyrosine residue nitration, which is also a consequence of increased ROS, facilitates proteasomal degradation, thereby accelerating protein turnover [30,42,43]. SOD1, also known as Cu/Zn-SOD, is located particularly in the cytoplasm but, also, in the nucleus, microsomes and the mitochondrial intermembrane space [44]. Alterations in the SOD1 expression have been linked to neurodegenerative diseases like amyotrophic lateral sclerosis [45]. In cerebrospinal fluid samples of NPC1 patients, SOD1 expression was higher than in unaffected individuals [23]. Similar results were obtained in the murine NPC1 BALB/c mice model, wherein an increased mRNA expression was observed [6]. Of note, a study conducted using NPC1-deficient fibroblasts reported reduced, instead of increased, SOD1 [26], which may be an example of cell type-specific effects in distinct disease models, hence illustrating the need for models recapitulating cell types and tissues affected in the disease of interest. 

Downstream of SOD1 and SOD2, catalase is a key player in the cellular antioxidative defense system. It mediates the decomposition of hydrogen peroxide into water and molecular oxygen [46]. Previous studies in NPC1 models revealed a reduced catalase activity. To our knowledge, no data obtained from human neuronal cells are available so far. Instead, a yeast model, BALB/c mice and fibroblasts of five patients [13,37,47] were used in previous studies. In accordance with these, we revealed a lower gene expression of catalase in all NPC1-deficient cell lines. Noticeable, all NPC1-deficient cell lines nearly completely lacked the catalase protein, as demonstrated by Western blotting. For cancer cells, it has been shown that catalase was silenced by a prolonged exposure to ROS through epigenetic methylation in the CpG island II in the promotor region of catalase [48] through the transcriptional activator Oct-1 [49]. Other regulatory mechanisms have been described, involving the p53 protein, the tumor necrosis factor α (TNF-α) and the peroxisome proliferator-activated receptor γ (PPARγ) [50]. PPARγ is known to be crucial for the biogenesis of peroxisomes, and these organelles have been shown to be affected in NPC1 [47]. Although the mechanisms leading to the downregulation of catalase mRNA and protein expression remain to be elucidated, the catalase deficiency observed in the used human model system strongly suggests a contribution to the pathophysiology of NPC1. Furthermore, Busciglio and Yankner [51] demonstrated that the addition of catalase to primary mixed cultures of human neurons and astrocytes, established from gestation fetal Down syndrome and normal cerebral cortex, ameliorated neurodegeneration, whereas the SOD addition had no effect [51]. Consequently, a restitution of a physiological catalase level may counteract the neurodegeneration and, thus, represent an intervention strategy for NPC1.

## 4. Materials and Methods

### 4.1. Cell Culture and Differentiation of iPSC-Derived Neurons

Human neural progenitor cells (NPCs) were obtained, as recently described [32], from patient-specific induced pluripotent stem cells (iPSCs) carrying on the following mutations: the prevalent homozygous mutation c.3182 T > C, the compound heterozygous mutation c.1836 A > C/c.1628 delC and the mutation c.1180 T > C. NPCs were obtained from one clone per cell line. NPCs were expanded in proliferation medium containing Dulbecco’s modified Eagle’s medium (DMEM), 60% DMEM/F-12, 1X B27, 0.5% penicillin/streptomycin, 20-ng/mL FGF2 (Amsbio, Abingdon, UK) and 20-ng/mL EGF (Peprotech, Hamburg, Germany) on poly-l-ornithine-coated (15 µg/mL; Sigma-Aldrich, St. Louis, MO, USA)/laminin (10 µg/mL; Trevigen, Gaithersburg, MD, USA) dishes. For terminal differentiation, cells were cultured in differentiation medium containing DMEM, 60% DMEM/F-12 and 1X B27, 0.5% penicillin/streptomycin. Medium was replaced every 3 to 4 days over a period of at least 40 days. All cells were cultivated at 37 °C under 5% CO_2_. Differentiation of NPCs resulted in a mixed culture containing neurons, as well as glial cells [32,52,53].

### 4.2. Western Blot

Western blot was performed using whole-cell lysates. Therefore, cells were covered with RIPA buffer (containing mM: TRIS 20, NaCl 137, sodium deoxycholate 12 and EDTA 2, supplemented with 0.1% SDS, 1% Triton^®^ X-100 and 10% glycerol with cOmplete™ Mini EDTA-free Protease Inhibitor Cocktail (Roche Diagnostics GmbH, Mannheim, Germany) and left on ice under slight agitation for 30 min to obtain cell lysates. Lysates were centrifuged at 15,000× *g* for 25 min at 4 °C. Protein concentration of the supernatant was determined using the Pierce™ BCA Protein Assay Kit (Thermo Fisher Scientific, Waltham, MA, USA) according to the manufacturer´s manual. Samples were boiled for 5 min at 95 °C in 5x Laemmli buffer (125-mM TRIS, 20% glycerol, 2% SDS, 5% β-mercaptoethanol and 10% bromophenol blue) and subsequently centrifuged at 22,000× *g* for one minute at 4 °C. Using a Criterion™ Vertical Electrophoresis Cell with Criterion™ TGX Stain-Free™ Precast Gels (4–15%) (Bio-Rad Laboratories, Feldkirchen, Germany), the proteins were separated. The electrophoresis buffer contained 250-mM TRIS, 2-M glycine and 0.1% SDS. For Western blot, the Trans-Blot^®^ Turbo™ Transfer System with Trans-Blot^®^ Turbo™ Transfer Pack (Midi Format, 0.2-µm nitrocellulose; Bio-Rad Laboratories, Hercules, CA, USA) was used. Afterwards, the membranes were washed in TRIS-buffered saline (TBS), containing 20-mM TRIS and 137-mM NaCl (pH 7.5) for 5 min and blocked with 5% BSA (Carl Roth GmbH & Co. KG, Karlsruhe, Germany) in TBS supplemented with 0.1% Tween^®^ 20 (TBST) for one hour. For protein detection of catalase, nitro-tyrosine, SOD1 and SOD2 membranes were incubated with primary antibody solution (3% BSA in TBST) overnight at 4 °C and for one hour at room temperature for the detection of GAPDH and β-actin. Subsequently, membranes were washed three times with TBST and incubated for one hour with DyLight™ secondary antibody in the dark. All used antibodies are listed in Table 1. Finally, membranes were washed three times with TBST and once with TBS and air-dried. Precision Plus Protein Dual Xtra Standards (Bio-Rad Laboratories, Hercules, CA, USA) was used as a molecular weight marker. The Odyssey Infrared Imaging System (LI-COR Biosciences GmbH, Bad Homburg, Germany) was used to visualize and quantify the protein signals. For the analysis of catalase, SOD1 and SOD2, the expression of β-actin was used for normalization. For the Western blots with nitro-tyrosine, a ratio was calculated for the nitrated proteins located at the 18 or 22-kDa bands (nSOD) or 42-kDa band (GAPDH) and the SOD1 at 18 kDa, SOD2 at 22 kDa or GAPDH at 42 kDa.

### 4.3. Determination of ROS Level with DCF Fluorescence

Intracellular ROS level was measured using the DCF Cellular ROS Detection Assay Kit (Abcam, Cambridge, UK) according to the manufacturer’s protocol. In brief, cells were incubated with 0.6 µM of the oxidant-sensitive fluorescent dye 2′,7′-dichlorodihydrofluorescein diacetate (H_2_DCFDA) in phenol red-free media (phenol red-free DMEM, 1-mM pyruvate and 1% penicillin/streptomycin) for 5 min in the dark at 37 °C. After washing the cells twice with PBS, they were harvested using Accutase (Stemcell Technologies, Cologne, Germany) for 5 min. The reaction was stopped with phenol red-free media, and 1.5 µM propidium iodide (PI) was added to the cell suspension shortly before the measurement. Fluorescence was measured using the 485-nm excitation wavelength and 535-nm emission wavelength for the DCF determination and 493-nm excitation wavelength and 585-nm emission wavelength for PI by means of a FACSCalibur (BD, Heidelberg, Germany). Data were sampled using the CellQuest Pro software (BD, Heidelberg, Germany) and analyzed with the FCSalyzer 0.9.18-alpha. Based on a negative control, a baseline was established for DCF and PI for each cell line. Fluorescent cells exceeding basal levels were designated as positive cells. PI-negative and DCF-positive cells were defined as ROS-positive cells. An example of the DCF flow cytometry analysis is shown for the control, as well as the c.1180 T > C mutated cells, in Supplementary Figure S1. 

### 4.4. Determination of SOD Activity

The SOD activity was determined using the SOD Determination Kit (Sigma-Aldrich, St. Louis, MO, USA) according to the manufacturer’s protocol, as described recently [54]. Cells were washed with PBS, and cell lysates were obtained by incubation with SOD lysis buffer (0.1-M TRIS/HCl, 1% Triton^®^ X-100, 10% glycerol and 0.05% SDS supplemented with cOmplete™ Mini EDTA-free Protease Inhibitor Cocktail (Roche Diagnostics GmbH, Mannheim, Germany)) for 30 min on ice. Afterwards, cells were subjected to 5 freeze and thaw cycles using liquid nitrogen and room temperature water, as well as 10 ultrasonic pulses (560 W, 1x/s). The samples were centrifuged at 15,000× *g* for 25 min at 4 °C. Protein concentration of the supernatant was determined using the Pierce™ BCA Protein Assay Kit (Thermo Fisher Scientific, Waltham, MA, USA) according to the manual. Samples were placed in a 96-well plate with or without xanthinoxidase enzyme solution, and WST-1 working solution was added. The base value of the superoxide anions of the samples (A_base value_) and the maximal (A_max_) and minimal (A_min_) WST-1 conversion were determined. Cells were incubated for 20 min in the dark at 37 °C under agitation. Absorption was measured at 450 nm with a Spark^®^ plate reader and SparkControl Magellan software (Tecan, Männedorf, switzerland). The SOD activity was calculated as follows: SOD activity (inhibition rate %) = ((A_max_ − A_min_) − (A_sample_ − A_base value_))/(A_max_ − A_min_) × 100. This value was normalized to the protein concentration of the sample. 

### 4.5. RT-qPCR

For reverse transcription quantitative real-time polymerase chain reaction (RT-qPCR), the cells were harvested with Accutase, and the pellet was resuspended in PBS. The RNA isolation was done with the Quick-RNA^TM^ Mini-Prep Kit (Zymo Research Europe GmbH, Freiburg, Germany) according to the manufacturer’s protocol. cDNA was obtained using the QuantiTect Reverse Transcription Kit (Qiagen, Hilden, Germany). PCR was performed using the FastStart DNA SYBRGreen Plus Kit (Roche Diagnostics GmbH, Mannheim, Germany) according to the manufacturer’s instructions. A LightCycler Nano (Roche Diagnostics GmbH, Mannheim, Germany) in combination with the LightCycler Nano 1.1 software or the Rotor Gene Q (Qiagen, Hilden, Germany) and the Rotor-Gene Q Series Software version 2.3.1 (Qiagen, Hilden, Germany) was used to quantify mRNA transcription. The following cycling parameters were used: initial denaturation at 95 °C for 600 s, 40 cycles of 20 s at 95 °C for denaturation, annealing for 20 s with the primer-specific annealing temperature and 23 s at 72 °C for extension. A melting curve was obtained using 65 °C for 60 s and 95 °C for 20 s to analyze the integrity of the PCR products. The amount of mRNA of the genes of interest was normalized to the reference gene YWHAZ. All samples were run in technical duplicates. Relative changes in mRNA amounts were calculated using Δ*C*_t_ values by means of the Pfaffl method [55]. For forward and reverse primer sequences, see Table 1. 

### 4.6. Antibodies and Primer

### 4.7. Statistical Analysis

The number of individual experiments was given as “*n*”. Individual experiments were at least obtained from three independent cell cultures. Analysis of the data was carried out with GraphPad Prism 6.07 (GraphPad Software Inc., San Diego, CA, USA). Data were given as mean ± SEM. Data were tested for normal distribution using D’Agostino-Pearson normality test. Normally distributed data were tested for statistical significance using ordinary one-way ANOVA test and Holm-Sidak’s multiple comparisons test. For not normally distributed data, one-way ANOVA using the Kruskal-Wallis test and Dunnett’s multiple comparison test were used to determine statistical significance. *p*-values < 0.05 were considered statistically significant, with * = *p* < 0.05, * = *p* < 0.01 and *** = *p* < 0.001.

## 5. Conclusions

In summary, we demonstrated for the first time that neuronal cells derived from NPC1 patient-specific induced pluripotent stem cells suffer from oxidative stress. Higher ROS levels, determined by DCF fluorescence, as well as changed amounts of nitrated proteins, indicate oxidative stress in all NPC1-deficient cell lines. Unexpectedly, we did not observe any alterations in mRNA or protein levels for SOD1 and SOD2. Still, we found alterations in the cellular antioxidative defense system. The protein and mRNA expression consistently displayed a massive reduction of catalase in NPC1-deficient cells. As catalase acts as a key player of the antioxidative defense system, we conclude that the lack of catalase contributes to elevated ROS levels observed in NPC1-deficient cells. A restitution of a physiological catalase level might display an intervention strategy to rescue NPC1-deficient cells from the repercussions of oxidative stress contributing to the neurodegeneration observed in NPC1.

## Figures and Tables

**Table 1:** List of antibodies, used dilutions and primer used for qRT-PCR.

Antibodies Used for Western Blot		
**Antibody**	**Dilution**	**Company**
Catalase; rabbit IgG	1:1000	Cell Signaling Technology, Danvers, MA, USA
nitro-tyrosine; mouse IgG2b	1:1000	Abcam, Cambridge, UK
SOD1; rabbit IgG	1:10,000	Abcam, Cambridge, UK
SOD2; rabbit IgG	1:1000	Cell Signaling Technology, Danvers, MA, USA
GAPDH; rabbit IgG	1:10,000	Abcam, Cambridge, UK
β-actin; mouse IgG	1:10,000	Sigma-Aldrich, St. Louis, MO, USA
**Primer**		
**Target**	**Forward/Reverse Primers (5′-3′)**	
SOD1	AGGCCCCTTAACTCATCT/CTACAGGTACTTTAAAGCAACTCT	
SOD2	GCACTAGCAGCATGTTGAGC/GCGTTGATGTGAGGTTCCAG	
Catalase	TTTCCCAGGAAGATCCTGAC/ACCTTGGTGAGATCGAATGG	
YWHAZ	GTCTGTAACTGAGCAAGGAGC/CTCTGCTTGTGAAGCATTGGG	

## Supplementary Materials

The following are available online at https://www.mdpi.com/1422-0067/21/20/7667/s1.

## References

[1] Vanier M.T. (2010). Niemann-Pick disease type C. Orphanet J. Rare Dis..

[2] Spiegel R., Raas-Rothschild A., Reish O., Regev M., Meiner V., Bargal R., Sury V., Meir K., Nadjari M., Hermann G. (2009). The clinical spectrum of fetal Niemann-Pick type C. Am. J. Med. Genet. Part A.

[3] Marí M., Caballero F., Colell A., Morales A., Caballeria J., Fernandez A., Enrich C., Fernandez-Checa J.C., García-Ruiz C. (2006). Mitochondrial free cholesterol loading sensitizes to TNF- and Fas-mediated steatohepatitis. Cell Metab..

[4] Yu W., Gong J.S., Ko M., Garver W.S., Yanagisawa K., Michikawa M. (2005). Altered cholesterol metabolism in Niemann-Pick type C1 mouse brains affects mitochondrial function. J. Biol. Chem..

[5] Reddy J.V., Ganley I.G., Pfeffer S.R. (2006). Clues to neuro-degeneration in Niemann-Pick type C disease from global gene expression profiling. PLoS ONE.

[6] Kennedy B.E., LeBlanc V.G., Mailman T.M., Fice D., Burton I., Karakach T.K., Karten B. (2013). Pre-symptomatic activation of antioxidant responses and alterations in glucose and pyruvate metabolism in Niemann-Pick Type C1-deficient murine brain. PLoS ONE.

[7] Torres S., Matías N., Baulies A., Nuñez S., Alarcon-Vila C., Martinez L., Nuño N., Fernandez A., Caballeria J., Levade T. (2017). Mitochondrial GSH replenishment as a potential therapeutic approach for Niemann Pick type C disease. Redox Biol..

[8] Vázquez M.C., Balboa E., Alvarez A.R., Zanlungo S. (2012). Oxidative stress: A pathogenic mechanism for Niemann-Pick type C disease. Oxid. Med. Cell. Longev..

[9] Murphy M.P. (2009). How mitochondria produce reactive oxygen species. Biochem. J..

[10] Schieber M., Chandel N.S. (2014). ROS Function in Redox Signaling and Oxidative Stress. Curr. Biol..

[11] Lin M.T., Beal M.F. (2006). Mitochondrial dysfunction and oxidative stress in neurodegenerative diseases. Nature.

[12] Uttara B., Singh A.V., Zamboni P., Mahajan R.T. (2009). Oxidative stress and neurodegenerative diseases: A review of upstream and downstream antioxidant therapeutic options. Curr. Neuropharmacol..

[13] Zampieri S., Mellon S.H., Butters T.D., Nevyjel M., Covey D.F., Bembi B., Dardis A. (2009). Oxidative stress in NPC1 deficient cells: Protective effect of allopregnanolone. J. Cell. Mol. Med..

[14] Klein A., Maldonado C., Vargas L.M., Gonzalez M., Robledo F., Perez de Arce K., Muñoz F.J., Hetz C., Alvarez A.R., Zanlungo S. (2011). Oxidative stress activates the c-Abl/p73 proapoptotic pathway in Niemann-Pick type C neurons. Neurobiol. Dis..

[15] Kennedy B.E., Madreiter C.T., Vishnu N., Malli R., Graier W.F., Karten B. (2014). Adaptations of energy metabolism associated with increased levels of mitochondrial cholesterol in Niemann-Pick type C1-deficient cells. J. Biol. Chem..

[16] Chung C., Puthanveetil P., Ory D.S., Lieberman A.P. (2016). Genetic and pharmacological evidence implicates cathepsins in Niemann-Pick C cerebellar degeneration. Hum. Mol. Genet..

[17] Vilaça R., Barros I., Matmati N., Silva E., Martins T., Teixeira V., Hannun Y.A., Costa V. (2018). The ceramide activated protein phosphatase Sit4 impairs sphingolipid dynamics, mitochondrial function and lifespan in a yeast model of Niemann-Pick type C1. Biochim. Biophys. Acta Mol. Basis Dis..

[18] Marín T., Contreras P., Castro J.F., Chamorro D., Balboa E., Bosch-Morató M., Muñoz F.J., Alvarez A.R., Zanlungo S. (2014). Vitamin E dietary supplementation improves neurological symptoms and decreases c-Abl/p73 activation in Niemann-Pick C mice. Nutrients.

[19] Smith D., Wallom K.-L., Williams I.M., Jeyakumar M., Platt F.M. (2009). Beneficial effects of anti-inflammatory therapy in a mouse model of Niemann-Pick disease type C1. Neurobiol. Dis..

[20] Porter F.D., Scherrer D.E., Lanier M.H., Langmade S.J., Molugu V., Gale S.E., Olzeski D., Sidhu R., Dietzen D.J., Fu R. (2010). Cholesterol oxidation products are sensitive and specific blood-based biomarkers for Niemann-Pick C1 disease. Sci. Transl. Med..

[21] Alvarez A.R., Klein A., Castro J., Cancino G.I., Amigo J., Mosqueira M., Vargas L.M., Yévenes L.F., Bronfman F.C., Zanlungo S. (2008). Imatinib therapy blocks cerebellar apoptosis and improves neurological symptoms in a mouse model of Niemann-Pick type C disease. FASEB J..

[22] Dominko K., Dikic D., Hecimovic S. (2020). Enhanced activity of superoxide dismutase is a common response to dietary and genetically induced increased cholesterol levels. Nutr. Neurosci..

[23] Cologna S.M., Jiang X.-S., Backlund P.S., Cluzeau C.V.M., Dail M.K., Yanjanin N.M., Siebel S., Toth C.L., Jun H.-s., Wassif C.A. (2012). Quantitative proteomic analysis of Niemann-Pick disease, type C1 cerebellum identifies protein biomarkers and provides pathological insight. PLoS ONE.

[24] Fu R., Yanjanin N.M., Bianconi S., Pavan W.J., Porter F.D. (2010). Oxidative stress in Niemann-Pick disease, type C. Mol. Genet. Metab..

[25] Ribas G.S., Pires R., Coelho J.C., Rodrigues D., Mescka C.P., Vanzin C.S., Biancini G.B., Negretto G., Wayhs C.A.Y., Wajner M. (2012). Oxidative stress in Niemann-Pick type C patients: A protective role of N-butyl-deoxynojirimycin therapy. Int. J. Dev. Neurosci..

[26] Woś M., Szczepanowska J., Pikuła S., Tylki-Szymańska A., Zabłocki K., Bandorowicz-Pikuła J. (2016). Mitochondrial dysfunction in fibroblasts derived from patients with Niemann-Pick type C disease. Arch. Biochem. Biophys..

[27] Rauniyar N., Subramanian K., Lavallée-Adam M., Martínez-Bartolomé S., Balch W.E., Yates J.R. (2015). Quantitative Proteomics of Human Fibroblasts with I1061T Mutation in Niemann-Pick C1 (NPC1) Protein Provides Insights into the Disease Pathogenesis. Mol. Cell. Proteom..

[28] Trilck M., Hübner R., Seibler P., Klein C., Rolfs A., Frech M.J. (2013). Niemann-Pick type C1 patient-specific induced pluripotent stem cells display disease specific hallmarks. Orphanet J. Rare Dis..

[29] Gilgun-Sherki Y., Melamed E., Offen D. (2001). Oxidative stress induced-neurodegenerative diseases: The need for antioxidants that penetrate the blood brain barrier. Neuropharmacology.

[30] Buchczyk D.P., Grune T., Sies H., Klotz L.-O. (2003). Modifications of glyceraldehyde-3-phosphate dehydrogenase induced by increasing concentrations of peroxynitrite: Early recognition by 20S proteasome. Biol. Chem..

[31] Peter F., Trilck M., Rabenstein M., Rolfs A., Frech M.J. (2017). Dataset in support of the generation of Niemann-Pick disease Type C1 patient-specific iPS cell lines carrying the novel NPC1 mutation c.1180TC or the prevalent c.3182TC mutation—Analysis of pluripotency and neuronal differentiation. Data Brief.

[32] Trilck M., Peter F., Zheng C., Frank M., Dobrenis K., Mascher H., Rolfs A., Frech M.J. (2017). Diversity of glycosphingolipid GM2 and cholesterol accumulation in NPC1 patient-specific iPSC-derived neurons. Brain Res..

[33] Beckman J.S. (1996). Oxidative damage and tyrosine nitration from peroxynitrite. Chem. Res. Toxicol..

[34] Pacher P., Beckman J.S., Liaudet L. (2007). Nitric oxide and peroxynitrite in health and disease. Physiol. Rev..

[35] Xiao G.G., Nel A.E., Loo J.A. (2005). Nitrotyrosine-modified proteins and oxidative stress induced by diesel exhaust particles. Electrophoresis.

[36] Miao L., St Clair D.K. (2009). Regulation of superoxide dismutase genes: Implications in disease. Free Radic. Biol. Med..

[37] Vilaça R., Silva E., Nadais A., Teixeira V., Matmati N., Gaifem J., Hannun Y.A., Sá Miranda M.C., Costa V. (2014). Sphingolipid signalling mediates mitochondrial dysfunctions and reduced chronological lifespan in the yeast model of Niemann-Pick type C1. Mol. Microbiol..

[38] MacMillan-Crow L.A., Thompson J.A. (1999). Tyrosine modifications and inactivation of active site manganese superoxide dismutase mutant (Y34F) by peroxynitrite. Arch. Biochem. Biophys..

[39] Yamakura F., Taka H., Fujimura T., Murayama K. (1998). Inactivation of human manganese-superoxide dismutase by peroxynitrite is caused by exclusive nitration of tyrosine 34 to 3-nitrotyrosine. J. Biol. Chem..

[40] Kim S.-J., Lee B.-H., Lee Y.-S., Kang K.-S. (2007). Defective cholesterol traffic and neuronal differentiation in neural stem cells of Niemann-Pick type C disease improved by valproic acid, a histone deacetylase inhibitor. Biochem. Biophys. Res. Commun..

[41] Huang Y., He T., Domann F.E. (1999). Decreased expression of manganese superoxide dismutase in transformed cells is associated with increased cytosine methylation of the SOD2 gene. DNA Cell Biol..

[42] Gow A.J., Duran D., Malcolm S., Ischiropoulos H. (1996). Effects of peroxynitrite-induced protein modifications on tyrosine phosphorylation and degradation. FEBS Lett..

[43] Matata B.M., Galiñanes M. (2002). Peroxynitrite is an essential component of cytokines production mechanism in human monocytes through modulation of nuclear factor-kappa B DNA binding activity. J. Biol. Chem..

[44] Okado-Matsumoto A., Fridovich I. (2001). Subcellular distribution of superoxide dismutases (SOD) in rat liver: Cu,Zn-SOD in mitochondria. J. Biol. Chem..

[45] Valentine J.S., Hart P.J. (2003). Misfolded CuZnSOD and amyotrophic lateral sclerosis. Proc. Natl. Acad. Sci. USA.

[46] Kirkman H.N., Gaetani G.F. (2007). Mammalian catalase: A venerable enzyme with new mysteries. Trends Biochem. Sci..

[47] Schedin S., Sindelar P.J., Pentchev P., Brunk U., Dallner G. (1997). Peroxisomal Impairment in Niemann-Pick Type C Disease. J. Biol. Chem..

[48] Min J.Y., Lim S.-O., Jung G. (2010). Downregulation of catalase by reactive oxygen species via hypermethylation of CpG island II on the catalase promoter. FEBS Lett..

[49] Quan X., Lim S.-O., Jung G. (2011). Reactive oxygen species downregulate catalase expression via methylation of a CpG island in the Oct-1 promoter. FEBS Lett..

[50] Kodydková J., Vávrová L., Kocík M., Žák A. (2014). Human catalase, its polymorphisms, regulation and changes of its activity in different diseases. Folia Biol..

[51] Busciglio J., Yankner B.A. (1995). Apoptosis and increased generation of reactive oxygen species in Down’s syndrome neurons in vitro. Nature.

[52] Rabenstein M., Peter F., Joost S., Trilck M., Rolfs A., Frech M.J. (2017). Decreased calcium flux in Niemann-Pick type C1 patient-specific iPSC-derived neurons due to higher amount of calcium-impermeable AMPA receptors. Mol. Cell. Neurosci..

[53] Peter F., Rost S., Rolfs A., Frech M.J. (2017). Activation of PKC triggers rescue of NPC1 patient specific iPSC derived glial cells from gliosis. Orphanet J. Rare Dis..

[54] Peskin A.V., Winterbourn C.C. (2017). Assay of superoxide dismutase activity in a plate assay using WST-1. Free Radic. Biol. Med..

[55] Pfaffl M.W. (2001). A new mathematical model for relative quantification in real-time RT-PCR. Nucleic Acids Res..

