# Supplementary material for: Oxidative Stress and Alterations in the Antioxidative Defense System in Neuronal Cells Derived from NPC1 Patient-Specific Induced Pluripotent Stem Cells
Source: Int J Mol Sci. 2020 Oct 16;21(20):7667. doi: 10.3390/ijms21207667 (PMC7593914; doi:10.3390/ijms21207667)
Supplement: Supplementary file 1 [file ijms-21-07667-s001.zip › ijms-896265-supplementayr-for conversion/Suppl Figure 1_.pdf]

International Journal of *Molecular Sciences*

MDPI

Supplementary Figure 1

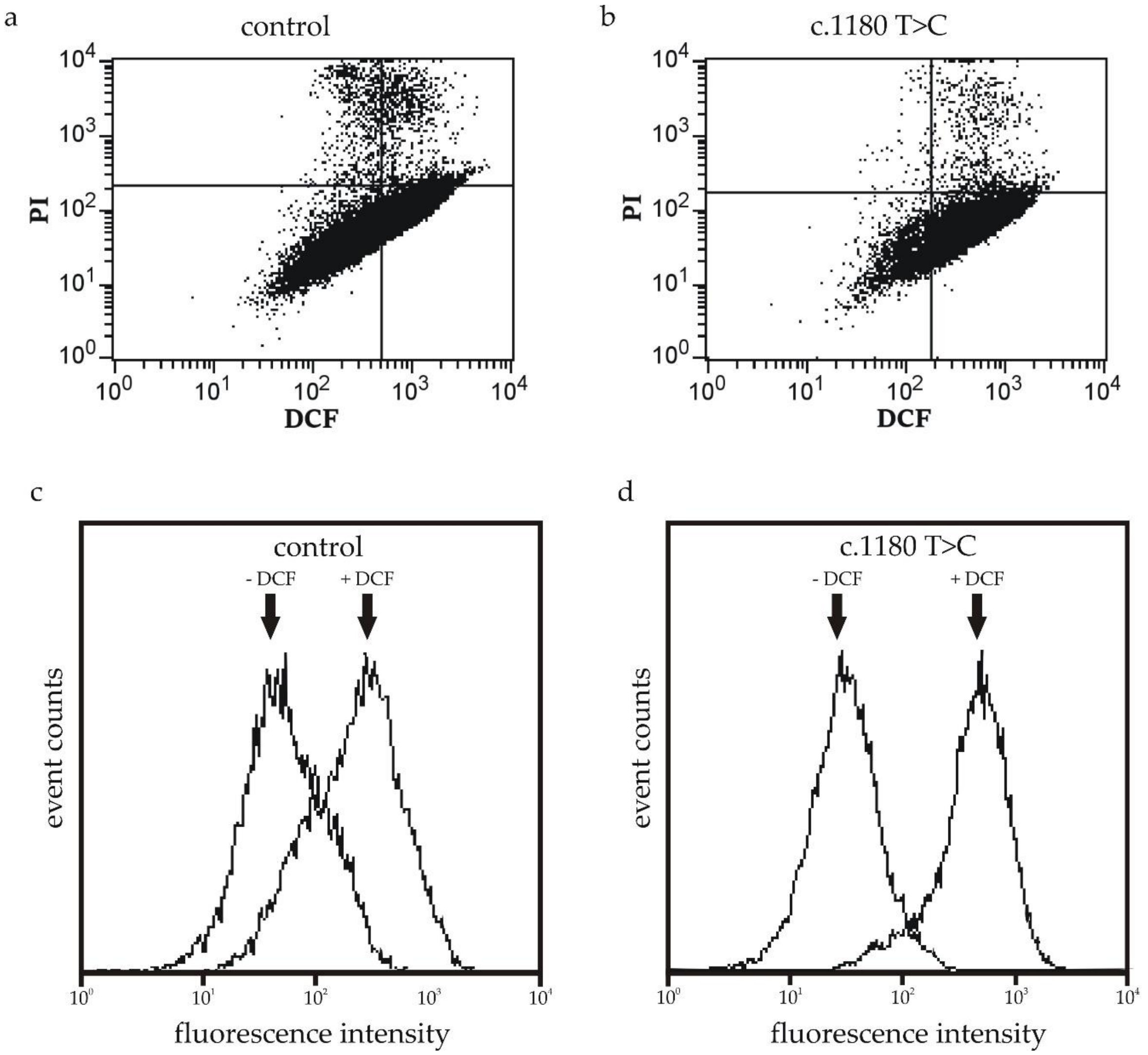

Supplementary Figure 1: Example of FACS analysis of ROS level by DCF fluorescence. (a) Dot plot of control cells and (b) of mutated cells simultaneously stained with propidium iodide (PI) and DCF. Lower left quadrant contains double negative cells. Lower right quadrant displays DCF positive cells and PI negative cells. This population reflects cells suffering from oxidative stress (OS). Upper left quadrant comprises PI positive cells and thus necrotic/apoptotic cells. Upper right quadrant involves PI and DCF positive cells. (c) Histogram of fluorescence distribution of control cells and (d) mutated cells. Cells not treated with DCF (-DCF) were used as negative control to set the cut off for the fluorescence analysis of cells treated with DCF (+DCF). For both examples one can observe the right shift of the DCF fluorescence, indicating cells suffering from OS. Noticeably, the shift to a higher fluorescence intensity is more pronounced in the NPC1-deficient cell line:

© 2019 by the authors. Submitted for possible open access publication under the terms and conditions of the Creative Commons Attribution (CC BY) license (http://creativecommons.org/licenses/by/4.0/).
