# Supplementary material for: Oxidative Stress and Alterations in the Antioxidative Defense System in Neuronal Cells Derived from NPC1 Patient-Specific Induced Pluripotent Stem Cells
Source: Int J Mol Sci. 2020 Oct 16;21(20):7667. doi: 10.3390/ijms21207667 (PMC7593914; doi:10.3390/ijms21207667)
Supplement: Supplementary file 1 [file ijms-21-07667-s001.zip › ijms-896265-supplementayr-for conversion/Suppl Figure 2_.pdf]

International Journal of *Molecular Sciences*

Supplementary Figure 2

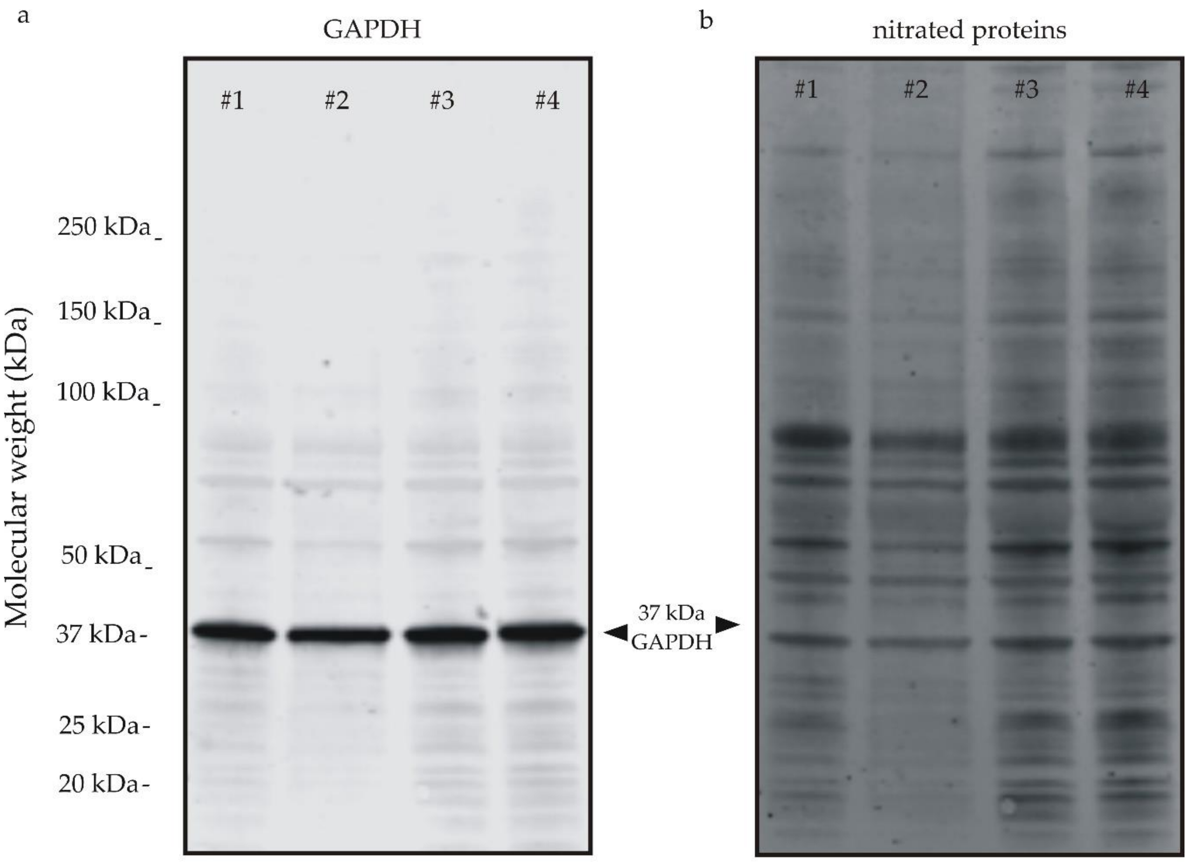

#1 = control #2 = c.3182 T>C

#3 = c.1836 A>C / c.1628delC #4 = c.1180 T>C

Supplementary Figure 2: Example of Western Blot to determine the ratio of nitrated protein
(a) Example of a Western Blot analysis to determine the amount of GAPDH in total cell lysate and (b) of the amount of nitrated proteins. Band located at 37 kDa in (b) were assumed to reflect nitrated GAPDH (nGAPDH). The ratio of nGAPDH and total amount of GAPDH, reflected by the bands located at 37 kDa in (a) was calculated to reveal differences between control cells and mutated cells.

© 2019 by the authors. Submitted for possible open access publication under the terms and conditions of the Creative Commons Attribution (CC BY) license (http://creativecommons.org/licenses/by/4.0/).
