# Supplementary material for: Oxidative Stress and Alterations in the Antioxidative Defense System in Neuronal Cells Derived from NPC1 Patient-Specific Induced Pluripotent Stem Cells
Source: Int J Mol Sci. 2020 Oct 16;21(20):7667. doi: 10.3390/ijms21207667 (PMC7593914; doi:10.3390/ijms21207667)
Supplement: Supplementary file 1 [file ijms-21-07667-s001.zip › ijms-896265-supplementayr-for conversion/Suppl Figure 3_.pdf]

International Journal of *Molecular Sciences*

MDPI

Supplementary Figure 3

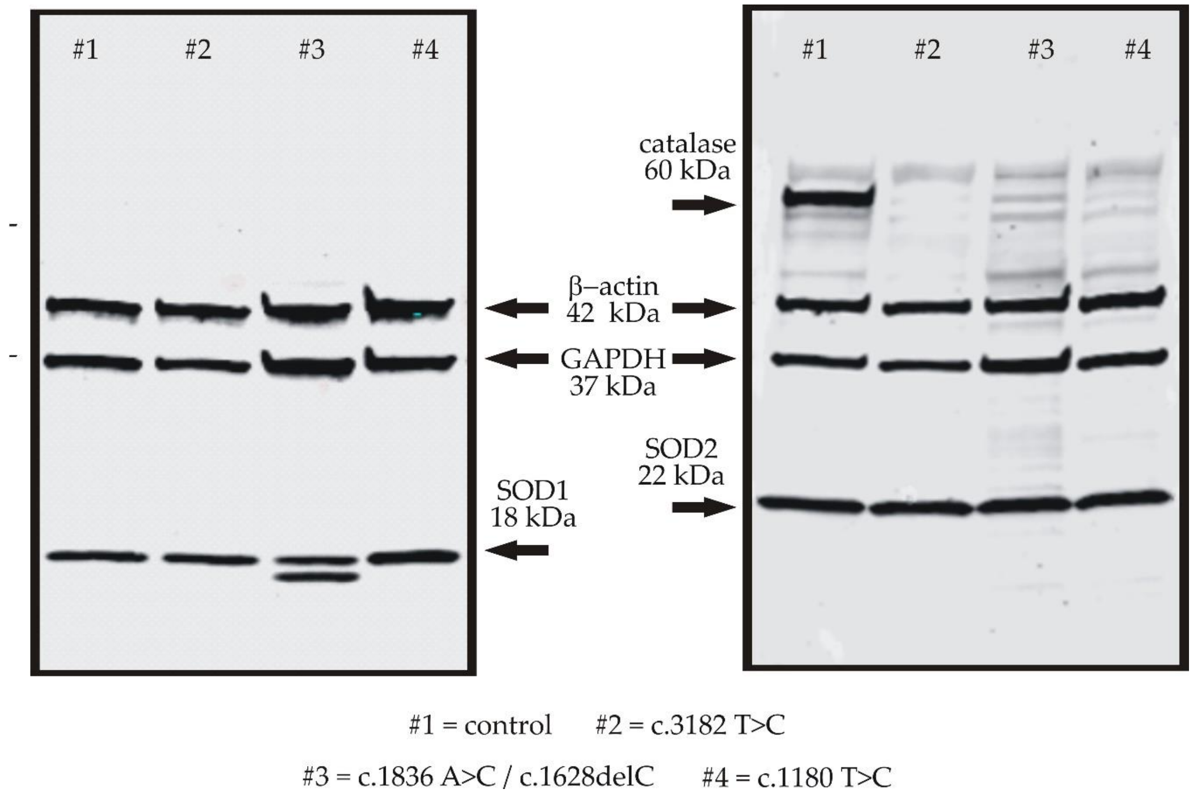

Supplementary Figure 3: Examples of Western Blots

Example of a Western Blot analysis to determine the amount of SOD1 (left panel), SOD2 and catalase (right panel). SOD2 and catalase were detected simultaneously on the same gels.

© 2019 by the authors. Submitted for possible open access publication under the terms and conditions of the Creative Commons Attribution (CC BY) license (http://creativecommons.org/licenses/by/4.0/).
